# Mapping the audit traces of interdisciplinary collaboration: bridging and blending between choreography and cognitive science

**DOI:** 10.1080/03080188.2017.1381226

**Published:** 2017-12-15

**Authors:** Philip Barnard, Scott deLahunta

**Affiliations:** ^a^ MRC Cognition and Brain Sciences Unit, Cambridge University, Cambridge, UK; ^b^ Deakin Motion Lab, Deakin University, Melbourne, Australia; ^c^ Centre for Dance Research, Coventry University, Coventry, UK; ^d^ Motion Bank, Hochschule Mainz University of Applied Sciences, Mainz, Federal Republic of Germany

**Keywords:** Interdisciplinary collaboration, long-term projects, contemporary dance, cognitive sciences, audit trace

## Abstract

Two long-term sci–art research projects are described and positioned in the broader conceptual landscape of interdisciplinary collaboration. Both projects were aimed at understanding and augmenting choreographic decision-making and both were grounded in research conducted within a leading contemporary dance company. In each case, the work drew upon methods and theory from the cognitive sciences, and both had a direct impact on the way in which the company made new work. In the synthesis presented here the concept of an audit trace is introduced. Audit traces identify how specific classes of knowledge are used and transformed not only within the arts or sciences but also when arts practice is informed by science or when arts practice informs science.

## Introduction

I thought, if we can find a way of starting to harness thought or start to understand a little bit more about how choreographic decisions are made, that’s absolutely going to feed into or build on aspects of choreographic thinking. Wayne McGregor (Barnard, deLahunta, and McGregor 2008)

This paper explores how and why sci–art relationships are set up, how they are supported and conducted and what outcomes they might indicate. It specifically asks what can be learned from such projects and, at a more fundamental level of enquiry, how such learning is recorded. In key respects the arts and sciences are both forms of enquiry and knowledge creation about the physical, social and cultural worlds we inhabit. The juxtaposition of different forms of enquiry then raises a point concerning the ‘bigger picture’ of our sci–art landscape: what forms of questioning and thinking might best promote and provoke interdisciplinary projects to debate deep assumptions that are intrinsic to the participating fields to begin with, and what outcomes can they create individually and together?

We explore these issues by drawing on two projects, Process and Concept Tracking (PACT) and Choreographic Thinking Tools (CTT), conducted during a decade-long research collaboration with the choreographer Wayne McGregor and his company Random Dance.^1^ The wider research programme involved investigating creativity in dance with specialists from other fields, including the cognitive and social sciences, and we briefly describe this background. We then present a framework, called the Bridging Model, for examining how representations of different types are used in the course of design activities. Although obviously different in form and content, processes of design lie at the heart of both science and art practices. Both can be thought of as using different kinds of knowledge to deliver some product into a social context, be that delivery from a science laboratory or from an art studio. The Bridging Model is our framework for analyzing these alternative forms. The model enables us to identify relevant sources of knowledge as well as the specific ways in which that knowledge is represented, used and transformed in iterations of design decision making. The identification of generic representations and processes adds value to interdisciplinary discourse. First it provides a common language for addressing science and arts practice. This in turn enables broader analyses of similarities and differences in what is learned and how it is learned in the conduct of different domains of science and arts practice as well as in interdisciplinary collaborations. Second, the generic language of design representations and processes enables us to provide explicit ‘audit traces’ not only of the use of knowledge within a discipline but also of how knowledge originating in one discipline comes to impact practice in another. We will follow the section on the Bridging Model with descriptions of the two projects, PACT and CTT, with the aim of revealing the audit traces that show the blending of science and art in these projects.

## Background

The wider research programme originated in a project called Choreography and Cognition (2003–2004), funded by Arts Council England and associated both with a residency for McGregor in the Experimental Psychology Department at Cambridge University and his subsequent creation of the dance piece AtaXia (Sadler’s Wells premiere 2004). McGregor and arts researcher deLahunta recruited a team of five scientists to join in a series of conversations related to the mental control of movement, its dysfunction and processes of dance creation. Each scientist was also given access to the dance company for the purposes of collecting data to answer questions concerning different facets of working with movement in a studio context. The topics included the multi-tasking of working memory in creation, movement perception, movement control, notations and the parts played by introspection and awareness during dance creation. In addition, a social anthropologist was recruited to study and assess the various interdisciplinary interactions (Leach 2006). The discussions leading to and surrounding these projects were a key contextual influence on McGregor’s creation of the dance piece AtaXia, in which he explored themes of incoherence and perturbation. The performance was presented to audiences as an outcome of a creative process that included the scientific investigations involved in Choreography and Cognition. The empirical work conducted by the various scientific teams also created concrete outcomes in the form of a number of academic articles and chapters directed at both the research communities associated with dance as well as the parent sciences (for a brief summary see McCarthy et al. 2006). The outputs are all referenced from the project website (http://www.choreocog.net).

**Figure:**
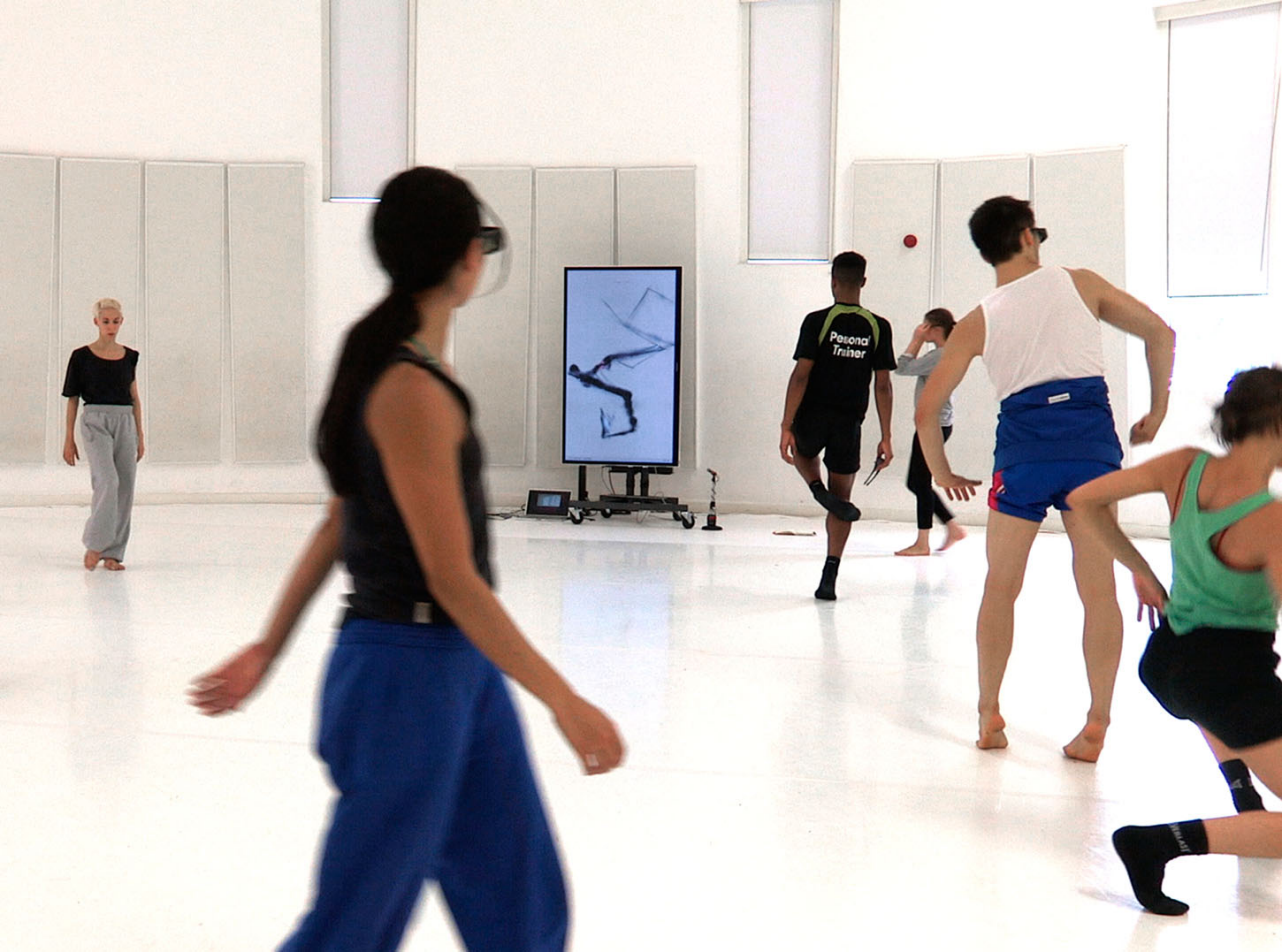
Wayne McGregor’s dance company developing the performance piece *Becoming*, using an artificial-intelligence system with a screen viewed with 3D glasses. Photo: David Bickerstaff, courtesy of Wellcome Collection.

The collaborative framework set up by McGregor and deLahunta was one in which everyone ‘could operate according to the requirements of his or her discipline … while never attempting a hybrid or common product’ (Leach 2006, 448).^2^ This framework was unique in a few key respects. There was a diversity of scientific perspectives and approaches involved and, as a result, the choreographer and dancers engaged with multiple forms of data collection including feedback about what was learned from those empirical observations. In effect, for a short period scientific activities were embedded in the day-to-day workings of a professional dance company. An essential element of this embedding was that the choreographer, the dancers and the scientists were very much equal partners, with everyone included in one form of dialogue or another, and all parties came away with material that they found useful for their own practice. Scientists wrote papers, dancers reported new perspectives on their decision making while working with movement, and McGregor crafted a work that was well received by audiences.^3^

However, while everyone gained something, the qualitative traces of what was gained were asymmetric. The day-to-day practice of science is conducive to formal audit, and understanding these audit traces in science are in part provided through the tradition of publication and references. Science papers, by their very nature, describe the background of a study, its rationale, its experimental design, how the data were analyzed and what conclusions follow from that analysis. Published scientific papers foster understanding and are intended to provide sufficient detail about process to allow a study to be replicated by others. The methods of artistic practice have conventionally not faced anything like the same requirements for audit. Narratives around historical influences in arts practice and stylistic evolutions over time are an integral part of the larger historical discourse. However, these narrative tracings, particularly in the case of choreography, tend to stop at the door of the studio. Indeed, other than the observation that McGregor found the research project and conversations leading up to AtaXia a productive *stimulus* for his making, exactly what he had taken from the dialogues and how he had used it at any level of his decision making were never really discussed or exposed. While there was a documented audit trace of the part played by the choreographic domain in the science output, vis-à-vis publication, the same could not be said for the traces of the science in the choreography: that remained rather ineffable. Our presentation of the Bridging Model below, followed by our discussion of PACT and CTT, seeks to redress this imbalance between the traditional audit traces in scientific work and current practice in the arts and in interdisciplinary collaborations.

## The bridging model and audit traces

**Figure 1.**
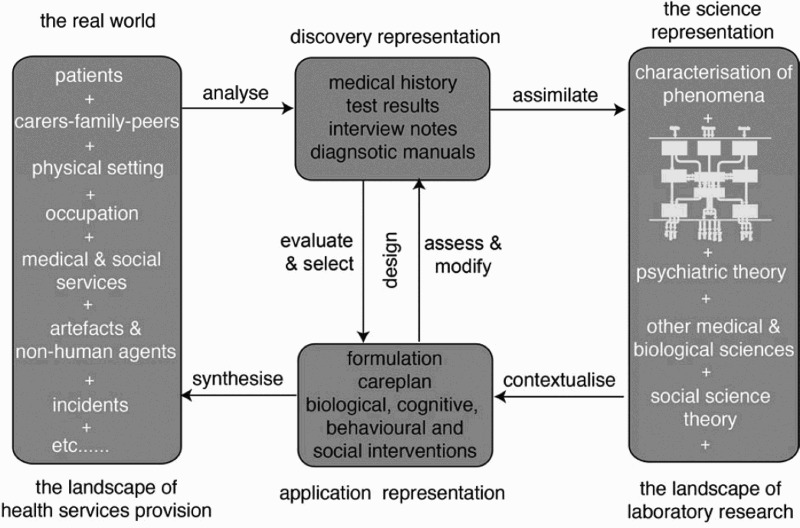
The model for bridging scientific knowledge into practical applications in the domain of clinical psychology (modified from Barnard 2004).

For the purposes of this discussion, we can consider both science and art as enterprises of design. Any process of design draws upon knowledge or experience and typically uses iterative techniques to develop some kind of product, such as a scientific theory, an artistic artefact, a performance or a text. Whatever the output, we can analyse what goes on, using a common language that identifies representations and processes that map one kind of representation to another. The approach taken here originated in earlier discussions about the properties of pure science and how it is applied in technology design (Barnard 1991; Long 1989). Its properties can usefully be introduced by summarizing a Bridging Model used for clinical psychology, shown in Figure 1, where the boxes index representations and the arrows index mapping processes (Barnard 2004). A key feature of this diagram is that we do not move from ideas to product in a single bound. Rather discovery and application representations act as bridges that mediate the iterative generation and evaluation of designs. This characterization acknowledges that there is a very rich and intricate representation of the real-world context in which a clinical application will be positioned (left-hand box). The designers working in this domain of application are clinical scientists. They will draw upon a range of scientific and technical knowledge as resources (right-hand box).^4^ In the course of the design process, they may develop specification documents, e.g. diagnostic tools (discovery representation, upper centre) to design a treatment intervention plan for a particular patient (application representation, lower centre) ready for use in the real-world context. In another example, not illustrated here but easy to imagine, an engineer may iterate through a design process in which they bring other discovery- and application-based tools to bear, like mathematics or computer models. In both cases, how engineers and clinicians approach the design enterprise will be a joint function of the representation of the real-world context for the application and the scientific knowledge on which they draw. In basic science, engineering and clinical sciences, the disciplines require detailed overt representations of both knowledge and how to use it (scientific papers, textbooks, manuals, lectures and much else). Such representations act as a ‘guarantee’ that work can be replicated and applied to good effect and safely.

**Figure 2.**
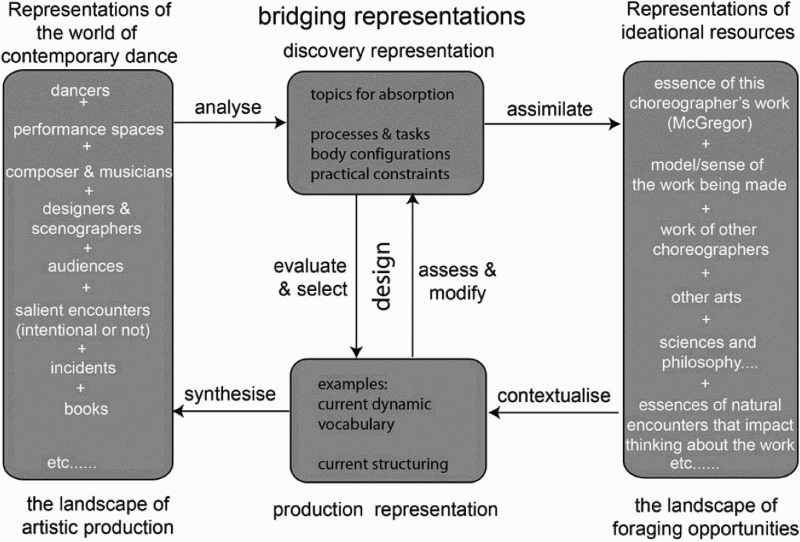
A model for bridging artistic knowledge into the making of contemporary choreography. Note that in artistic design processes, the landscape of foraging opportunities makes references to other disciplines. The discovery representation references the methods, tasks or constraints used in the creation of the production representation (e.g. the movement material for the new work), both are processes studied within the CTT project, while the knowledge sources and essences of natural encounters under ideational resources are topics explored in the PACT project. Both projects will be elaborated on in the following sections.

The eight labelled arrows in Figure 1 specify the kinds of thought processes the clinician or applied scientist must use in order to generate and apply their scientific knowledge. For example, they need to *analyse* the real world inhabited by a patient and then apply discovery methods (history taking, tests etc.) and *assimilate* the products of that into their broader scientific knowledge. Relevant knowledge needs to be *contextualized* and intersected with the products of the diagnostic tests to develop a care plan (one type of application representation) in which multiple considerations may need to be *synthesized* to accommodate the practical constraints associated with the particular patient’s lifestyle and social conditions. In this case, the specific care plans may be developed through an iterative process of design that takes place in the centre of the diagram. While clearly constituting a core element of practitioner expertise, these eight processes – even for scientific reasoning – are poorly studied and articulated. These processes arise out of *craft skills*, and the success of any design enterprise is critically dependent on them and on the ability of the practitioner to proceed iteratively from one side of the bridging model to the other; passing through different versions of discovery and representations. If one were to audit the adequacy of the science, then published papers would enable a reader to populate the relevant elements of the discovery representation, the science representation and the application representation and trace key aspects of the reasoning involved. The principal point to establish here is that this kind of framework is also applicable to the conduct and craft skills of artistic work.^5^ Figure 2 illustrates how the Bridging Model framework can capture the conduct of the design and production activities of Wayne McGregor, his company and artistic collaborators. The same basic framework would hold for other contemporary choreographers with whom the authors have worked or been associated, such as William Forsythe, Emio Greco|PC and Siobhan Davies. What would differ for each artist would be the specific representations within each of the boxes.

Mapping the work of artists into the same structure as that of scientists enables a direct comparison of audit traces for both domains and a means of thinking about interdisciplinary collaboration. It is, of course, vital to acknowledge similarities and differences between the conduct of arts and science projects. Science relies more heavily on representations that capture phenomena, facts and systematic accounts of them, while the arts draw on a relatively more open-ended range of sources of inspiration. Whereas scientific paradigms place emphasis on requirements to replicate and generalize, practices across the humanities, art and art scholarship are more likely to credit individual uniqueness and interpretation of meaning in social contexts.

In both cases, it is clear that the products of design processes in the sciences and in the arts can take a variety of forms, some more transitory than others. These forms are the result of a myriad of decisions or responses, themselves reactions to states of our social, physical and conceptual worlds. In both sciences and arts, decisions may emerge from fast intuitive thinking in the moment or from more deliberative mechanisms that are rather more open to reflection and study. The Bridging Model offers a conceptual schema for representing key properties and roles of the precursor states of decisions (including their basis in domain assumptions) and their wider role in such design processes. An audit trace makes explicit the part played by ideas and methods in the genesis and subsequent trajectory of design from the inception of a project to its conclusion. In most complex projects, a full audit trace would be of intractable complexity. However even within complex projects, key aspects of how the project draws on attributes of the social setting from which it arises and into which a project is delivered can be extracted. Likewise, key attributes of the deeper knowledge sources and bridging representations can be identified and probed to form an audit trace that is sufficient to support systematic discussion.

Importantly, the concept of an audit trace helps us focus on where highly specific processes are occurring at the intersections of ideas and methods that may not appear in the end product. This gives us the unique opportunity to establish and evaluate where and how interdisciplinary collaboration takes place, at the point where the audit trace of science application overlaps with the audit trace of art production. For example, we can see how discovery methods applied in the studio are assimilated to a scientific understanding of creative processes or how knowledge, perhaps in the form of a scientific theory, is contextualized by an artist in framing the form of an artwork. More detailed discussion of the use of the Bridging Model and concept of audit traces will follow in the two collaborative research project descriptions below. Both addressed McGregor’s wish expressed at the start of this chapter to ‘start to understand a little bit more about how choreographic decisions are made’ and to ‘build on aspects of choreographic thinking’. The PACT project, established a method of recording and annotating rationales used by artists to help them, researchers and commentators to better understand the underpinnings of choreographic decision-making. The CTT project explored how a science-based augmentation of choreographic practice can be designed. Together they provide sufficient substance for us to discuss not only how the Bridging Model supports the discovery of audit traces for a contemporary arts practice, but also how the audit traces can reveal the overlap between different disciplines working together collaboratively at the point where they productively intersect.

## Project 1: process and concept tracking

Observations in the studio enabled us to clearly see what McGregor was doing in sufficient detail to describe his methods (Kirsh et al. 2009) and go some way towards specifying the content of his studio-based discovery representation (see Figure 2), but much of the background knowledge and conceptual ideas that shaped his decision making there remained latent. In those studies of his methods, which involved extensive interviewing by a cognitive scientist, McGregor was highly articulate about his choreography and his ways of working collaboratively with his dancers and other artists. He also frequently gave interviews to journalists in connection to a production. These interviews, in conjunction with the performances, constitute the public face of his choreography. What these efforts do not typically expose are those parts of the creative process that begin well before McGregor and his dancers enter the studio to work together and may involve a range of decisions not shared with others. Discussions of these issues, coupled with the vision expressed in the quote at the start of this paper, led to the idea that it would be helpful to study how the choreographer’s thinking evolved in the gestation period of planning a new production and throughout its making to the point of public performance.

**Figure 3.**
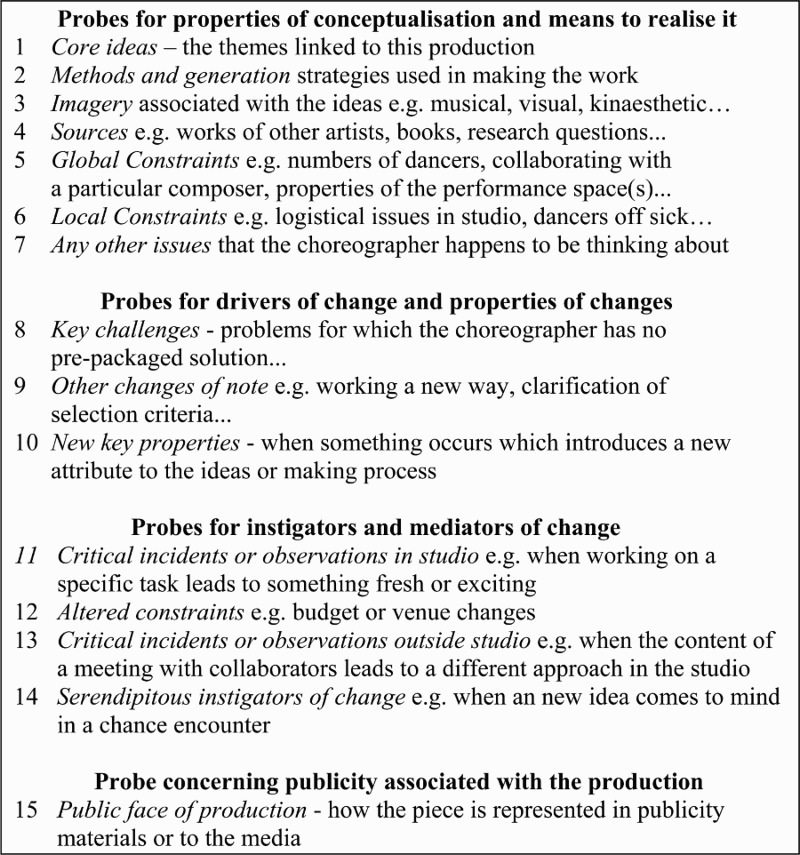
Categories of knowledge probed in PACT.

The objective of this strand of research was thus to explore this latent knowledge and identify at least some aspects of how it was used in the choreographer’s decision making. Initially using input from arts research and from research in cognitive science that had already explored a range of techniques for eliciting knowledge used in decision making (e.g. Cooke 1994), we identified 15 topics that it would be good to sample repeatedly in a structured interview format over the full-time course of making a choreographic work (Figure 3). Seven topics were concerned with the conceptual ideas associated with a piece and the means to realize them in performance. Three further topics concerned drivers and properties of change while four focused on the mediators of change. A very important feature of this project was that the same interview protocol was used to sample choreographic thinking across the full course of making; meaning that PACT interviews with McGregor were conducted at several key points throughout the process. Once the content of these interviews had been summarized in bullet form in a network of boxes on a sequence of coding sheets, something akin to a map of the specific sequence of choreographic thinking was relatively easy to inspect and use to index and access more detailed content in the far more extensive interview transcripts.

This procedure was given the title ‘Process And Concept Tracking’ or PACT and was initiated when a date for its opening performance was fixed. The interview sequence varied from production to production, but took place in two phases – prior to making and during making. In the case of the most recent production ATOMOS, which premiered at Sadler’s Wells in October 2013, eight interviews were conducted: five were conducted before the company came together in the studio and three after that, with the final one conducted on the actual day when the production opened.^6^ The core output of the procedure was a large corpus of interview material. In a first analysis, the authors reviewed the protocols together with the transcripts and extracted summary phrases for segments of dialogue. These were then assigned to one of the headings shown in Figure 3 and entered as bullet points in the relevant segment of a coding form for that interview. The content of these forms acted as a précis from which we could grasp what the central ideas were and what McGregor was doing with them. When changes occurred we could identify them as well as what drove and mediated them.^7^ The set of PACT forms for a production, and what was encoded on them, were the elements of the notation that enabled us to track or trace his thinking, decisions and the knowledge drawn upon to make them, moving backwards and forward in time. This is how an analysis of the ATOMOS PACT material can reveal traces of choreographic thinking.

The two examples in Table 1 illustrate how an underlying artistic intent receives expression and morphs over time in response both to the practical constraints of making and to the exploration of connections among the sets of ideas that happen to be in play at a particular point in the larger landscape of the trajectory of making. The full PACT transcripts can be considered to contain a reasonably sized ‘sample’ of choreographic thinking, not only of *what* is being thought, but also about how the larger decision space is managed over time. The PACT process acknowledges, and gives form to, rich sets of layered and interconnected artistic rationales. It shows how iterations of choreographic enquiries can be rendered explicit and support grounded inferences about these rationales. The basic output of PACT is a form of indexed and categorized transcript, and is therefore data that is amenable to scrutiny by those from different communities of practice who may wish to extract and infer different audit traces on the basis of their own conceptual filters. The overall PACT project was made possible because it was embedded in the work of the company over several productions, not just one. PACT was intended as a method of data collection about choreographic thinking, decision making and rationales to support the long-term objective as articulated by McGregor in the quote at the start of this article. The PACT project clearly enables the Bridging Model in Figure 2 to be populated with concrete traces of McGregor’s choreographic process, both in terms of difficult-to-extract craft skills occurring along the arrows, as well as filling the discovery and production representations with content. In Trace 1 in the table above, we see how his metaphorical use of the scientific properties of atoms come to shape his discovery and production representations (see centre of Bridging Model in Figure 2) guiding the development of movement material. It also shows how the mathematics of prime numbers is contextualized as a means of structuring the final performance. This puts artistic rationale on an equal level with science by providing the audit trace of iterative decision making toward the final production. Without the PACT process, for example, the mechanism of structuring would most probably have remained latent within the piece, as was the deep relationship among diverse forms of imagery that were all inter-related to the film Blade Runner.

**Table 1.** Traces of choreographic thinking in the production of ATOMOS.

Trace 1 A persistent concern of McGregor’s is his interest in essence associated with the concept of ‘qualia^8^.’ This was a central thread in conversations leading up to an earlier work (FAR, Sadler's Wells premiere 2010) and re-emerges in the first PACT for ATOMOS (May, Sadler's Wells premiere 2012) as the possible title of a new work, Quantum, inspired by physics. In December, Quantum was replaced by ATOMOS, which brings the specific property of ‘indivisibility’ into focus, one that also figured years before in the background projections for AtaXia (Sadler's Wells premiere 2004). By May, the idea of indivisibility is coupled with the notion of something having the property ‘uncuttable.’ Here he is contemplating exploring how the idea of uncuttability might work within his practice in the studio. We also see two **key ideas** coming together: ‘atoms’ and ‘growing’. In August, after two weeks in the studio, he has most of the ‘atoms’ – segments of movement material – he is looking for. Now the questions are about qualities and what is to be kept or discarded. In the final stages a set of prime numbers (mathematically indivisible) form the scaffolding of his decision making and subsequently determines the precise structuring of the piece.
Trace 2 Another thread traced across the ATOMOS process related to McGregor’s interest in using human data technology – in the studio, onstage and perhaps as a means of recording variation across all performances. Early on in pre-studio PACT (December 2012) McGregor’s thoughts focus on the connections between emotion, data and movement, including references to body data, states of arousal and invisibility. In February, the idea appears as a key challenge: how might it be realized practically and artistically? In May, it is being considered again but now as ‘body broadcasting,’ an ambitious new, yet specific, core idea that involved using known technology to measure the audience’s emotional response to the performance and using that data to influence what subsequently happens on stage. By the July session it is becoming a trickier, intimate question. In the rehearsal studio in early August it is back in the world of methods of making: what might you do with human data in the studio and how? The core idea persists and ultimately becomes a more tractable process of using that known technology to record his own dancers’ reactions to the film Blade Runner. This film was the key resource mapping into all aspects of this production, including imagery used in making and imagery used in the on-stage scenography. An essential theme in McGregor’s thinking related to how one might know whether or not an individual is an android ‘replicant’. Given pragmatic constraints, the data actually recorded from the dancers was ultimately used to derive and print abstract imagery onto the dancers’ costumes by Studio (2013). The collective on-stage imagery had a common but latent deep structure.

As the series of interviews progressed, it was clear that McGregor did not simply arrive at the interview to be probed. He increasingly came prepared, having thought about what he might say about individual topics within the protocol, and found that the process itself helped him frame his decision making. Partly in response to this observation, the potential educational benefits of a PACT process for students of choreography were explored in collaboration with Trinity Laban, a London-based conservatoire of music and contemporary dance. Pairs of students interviewed each other using the protocol across the course of their MA thesis project. This extended our exploration of the value of exposing and inspecting experiences of making and, through the very act of recording it, facilitating much of the groundwork for subsequently writing up and presenting their projects for examination. The ideas that the procedure could be both a means of data capture and *a tool for thought* for McGregor and the students was unforeseen at the outset of the work (deLahunta and Barnard forthcoming).

While the products of the PACT methodology offer a systematic grounding for generating intellectual audit traces of many strands of choreographic reasoning, use of knowledge and decision making that occur, it does so at a rather macroscopic level of analysis. However, the development of the PACT protocol and systematic probing took place overlapping with a related but different research programme titled ‘Choreographic Thinking Tools’ or CTT (McGregor et al. 2013). The CTT project began with two key questions: How can we uncover more about the kinds of intelligences (choreographic thinking) that are involved in contemporary dance making? How can we make this information available to choreographers in a format that could be of use in their practice? The project focused specifically on the phase of work in the studio where dancers are given tasks (discovery representations in terms of the Bridging Model) to generate movement material. What we will show in the following section is how the development of the CTT evolved through several iterations, illustrating a different form of audit trace, one that shows a clear connection to scientific data and theory, not as an inspiration for making but as a basis for augmenting choreographic decision-making in the studio.

## Project 2: choreographic thinking tools

**Figure 4.**
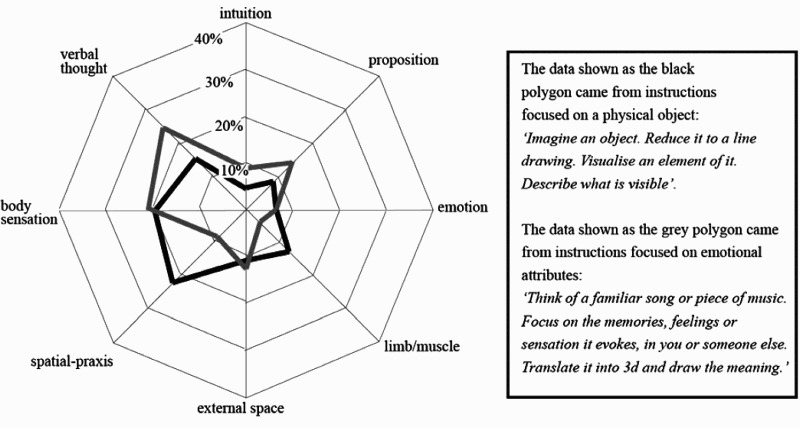
Illustrative data from one experience sampling study of eight professional dancers (May et al. 2011). At the apex of each spoke of the ‘spiders web’ arrangement is a description of a type of mental image (e.g. a verbal thought). The distance out from the centre captures the percentage of the ‘stops’ that an image on that spoke was reported. Shown here are data from instructions to draw or describe in movement the content of emotional imagery or imagery of a physical object. The key finding is that the pattern changes with the type of task McGregor asks them to solve. Dancers were often surprised by the low frequency of intuitive and emotional responses and the higher frequency of verbal thoughts.

The specific objective of this collaborative work was to establish how we might develop and use knowledge from the cognitive sciences to engage more fully the imaginations of the dancers when making new material. Initially the project drew on movement parsing work conducted during the Choreography and Cognition residency at Cambridge in 2004. These studies established some important parameters surrounding the considerable variability in how movement phrases are parsed. They also brought into sharp focus the potential value of using discussions and research feedback to bring into the dancers’ awareness aspects of their own practice that they themselves had not interrogated systematically (deLahunta and Barnard 2005; deLahunta et al. 2006). This was followed up in a whole series of empirical studies that used a method called experience sampling. McGregor himself has a very rich repertoire of studio practices he utilizes to develop a large body of movement material in the early stages of making. He employs a number of different methods that were also studied during the making of the choreography for DYAD 1909^9^ using an ethnographic methodology (see Kirsh et al. 2009). A key element of one method is to give his dancers tasks to make movement material, the instructions for which invoke mental imagery and a range of state transformations on those images.^10^ Such tasks were selected as a target for our experience sampling research, as illustrated in Figure 4.

The scientific methodology of experience sampling was used to probe what was going on in the minds of dancers while they were actually creating movement material. Two basic kinds of probes were used. One involved stopping dancers in the course of making and asking them to record key properties of the image or thought that went through their mind in the moment before they were stopped. In a number of iterations a range of tasks the company employed in their routine practice were compared. As the research progressed, the procedure was refined with student samples and professional dancers, leading up to a key study using the full company at Wayne McGregor | Random Dance (see May et al. 2011).

As a result of these studies new insights were generated about patterns of imagery and thinking in making, along with some insights into how they changed in response to the different forms of task instruction provided by the choreographer (right side of Figure 4). In the course of these studies, the dancers underwent lengthy periods in which they reflected individually and collectively about what was going on in bodies and minds whilst creating movement material using systematic data gathering protocols derived from cognitive theory. Some key revelations emerged through sharing, not just observations about movement, but also observations about the wide range of *mental strategies* that were called into play when solving the same movement problem. Once this variation is experienced and grasped, new possibilities for using a larger range mental strategies under intentional control became possible not only when manipulating mental images but also when translating properties of those images into movement.

**Figure 5.**
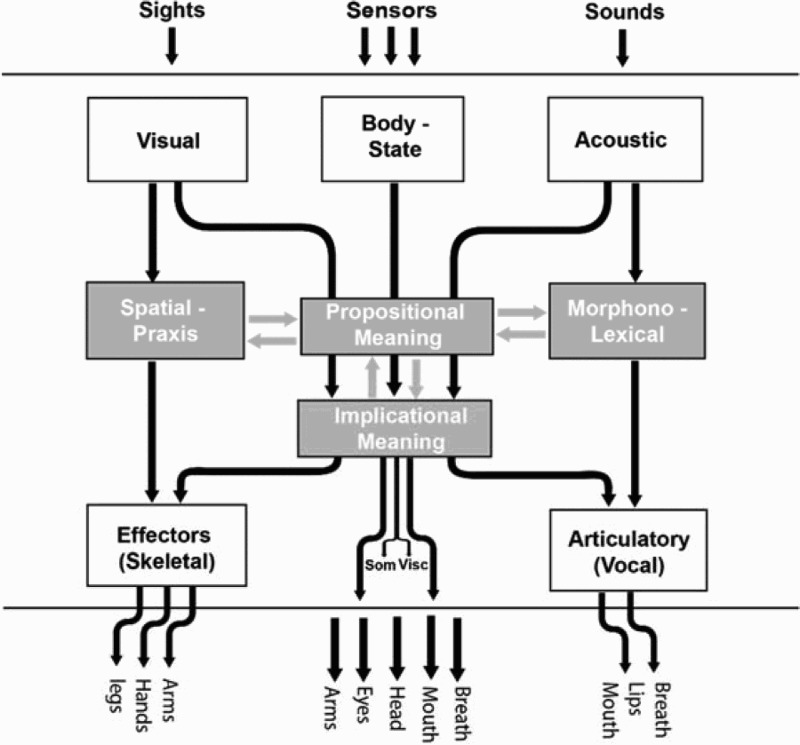
In the upper part of this illustration there are three sensory subsystems that initially transform sense data and pass it on in specific pathways to four cognitive subsystems, highlighted in grey. These cognitive subsystems support three internal mental dialogues or *interactions* (shown as double grey arrows) that generate mental imagery and, once formed, image content undergoes translation into overt actions either through effector subsystems that manage the control of skeletal effectors and vocal articulation or via somatic and visceral response systems.

These studies and the various instruments developed to support the data collection were based upon a theoretical framework from cognitive science called Interacting Cognitive Subsystems (ICS – Barnard and Teasdale 1991). This framework is illustrated in a simplified schematic form in Figure 5. It provided the definitions of mental images and senses of meaning both cognitive (‘propositional’ meaning) and cognitive–affective (‘implicational’ meanings that are formed by integrating multisensory sources with the products of processing propositional meanings). This theoretical framework brought with it two particularly advantageous features for applications to choreographic thinking. One was the inclusion in its architecture of mind of a *body state subsystem* of equal status to those subsystems specialized to handle material in our visual and acoustic landscapes. The other was the way in which it characterized the rich relationships between imagery of meanings, dynamic imagery of visual forms in the mind’s eye and imagery of auditory/verbal forms in the mind’s ear.

**Figure:**
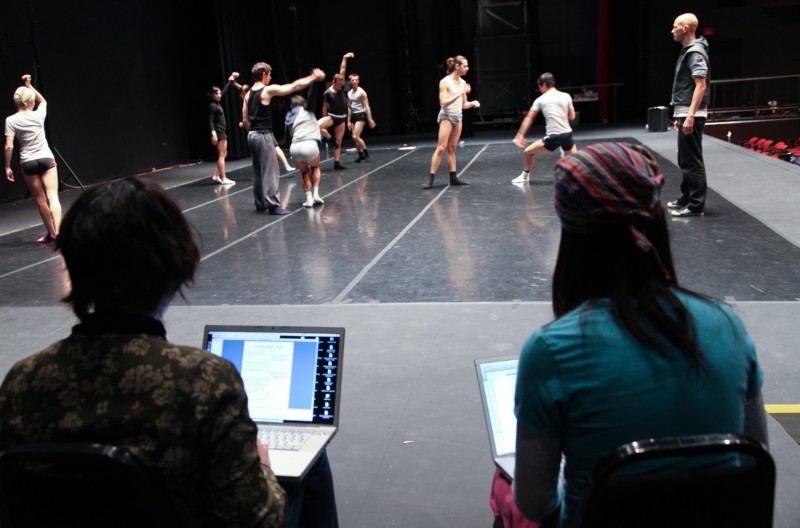
Wayne McGregor making *Dyad 1909* at the University of California at San Diego in 2009 while two researchers record the making process. Photo: Adrienne Hughes.

The patterns of tasking imagery as discussed in relation to Figure 4 can be mapped directly onto interactions among the four cognitive subsystems. Spatial-praxic^11^ imagery is what we experience when we generate images in the mind’s eye, while morphonolexical imagery is what we experience when we mentally generate verbal thoughts, sounds or music in the mind’s ear. Rather more intricate are interactions in semantic imagery. Here semantic imagery is seen as emerging out of an ongoing dialogue between intuitive, embodied and potentially emotional ‘implicational’ meanings on the one hand and the conceptual properties that form specific propositions on the other hand. The pathways highlighted as grey arrows in Figure 5 are purely mental dialogues that are important constituents of thought patterns in general and choreographic thinking in particular. There is much more detail that underpins this general picture. However, these descriptions should be sufficient to illustrate how we addressed the second element of the opening quote from McGregor – that is how both theory (Figure 5) and data (Figure 4) from cognitive science might ‘feed into or build on aspects of choreographic thinking*.*’

Against the background of experience sampling and other opportunities to meet, discuss and refine the theory, was a significant two-week residency in the USA in early 2010.^12^ Here McGregor, the dancers and the authors came together as equal partners to co-design a set of resources to help dancers extend not just physical techniques but also the mental and creative techniques they could use when making new material. This included a series of daily workshops over a week during which proposals were offered, tried in the studio and then revised in response to testing. Various materials were developed that integrated ideas about mind and movement derived from cognitive science with practical application by the dancers. The result was a refinement of some simple instruments for systematic note taking (a new discovery representation following the Bridging Model in Figure 2) and methods to help the dancers recognize how they were using mental imagery; how they were using transformations of image states; and how such imagery was translated into movement.

The end product is best characterized as a ‘process’ embedding a number of elements. Some elements, such as illustrations and strategies for attending to image content, could be traced directly back to the input from basic science; other elements, including specific transformations on images (e.g. ‘Imagine an object. Reduce it to a line drawing.’ – see Figure 5), were derived from resources that McGregor already used to frame his tasks; and yet others, such as strategies for concretizing abstractions, came from the dancers sharing their ‘how-I–did-it’ experiences. This CTT research and resulting ‘process’ offered McGregor insights into the use of imagery in movement creation which he could draw on for refining his method of deriving and offering tasks/exercises to the dancers. It helped the dancers develop cognitive skills of imagination as well as physical skills of movement expression.^13^ It was also envisioned to function as a kind of add-on to company class, to resource the dancers before entering into a particular phase of making with the view that they might train their ability working with various forms of imagery they might anticipate would come to them as a task exercise.^14^

**Figure 6.**
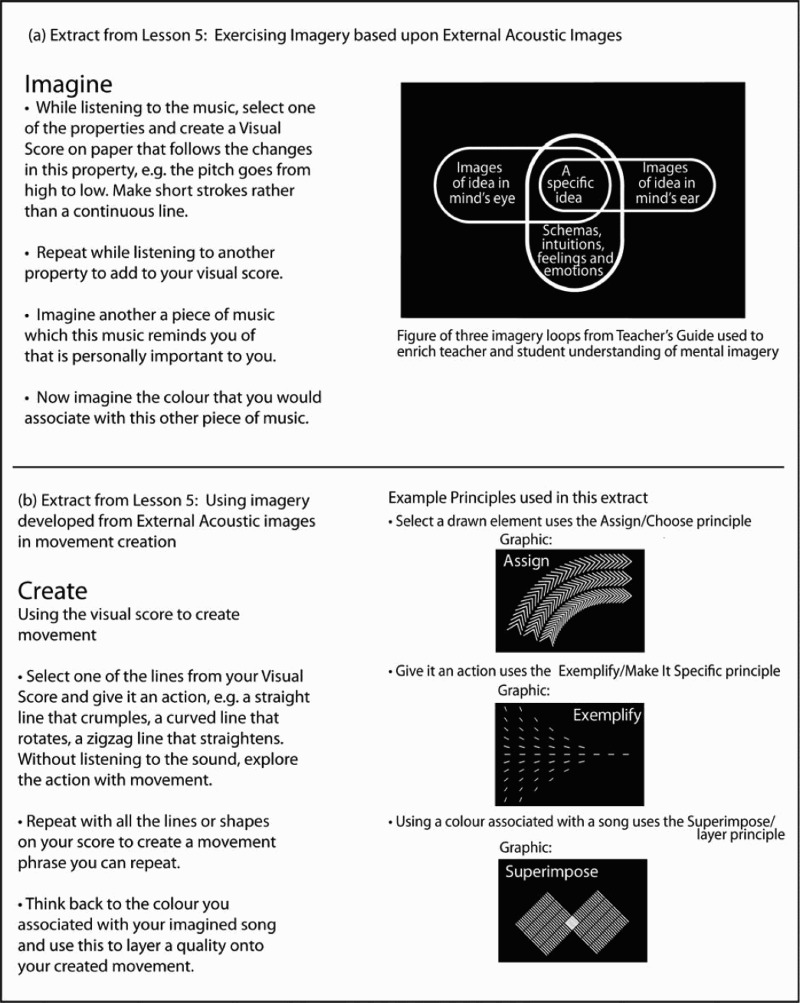
(a) An example of an imagery task and (b) a creation task along with illustrations of the principles used. Note the upper panel makes graphic reference to three imagery ‘loops’ in the mind. These three loops are a simplified form of the interactions shown in Figure 5, and their graphic form was designed to be compatible with the graphics created to illustrate principles derived from McGregor’s practice. Text is extracted from the Mind and Movement resource. Image Credits: Magpie Studio for lower panel and Philip Barnard for the upper panel.

Following on from the research and development work with the professional dancers, a grant was obtained from the Paul Hamlyn Foundation to develop an educational resource to support the teaching of creative skills to young dance students. The development of this resource was again an extended process of iterative development, testing and evaluation. The result was ‘Mind and Movement: Choreographic thinking tools’ McGregor, Barnard, and Dancers (2013), a boxed set containing several components: a teacher’s guide; twelve principles for working with images and movement (e.g. assign, exemplify, superimpose) printed on individual cards and a poster; four lesson plans each focusing on a different kind of stimulus for making and some guidance for developing solos, duets and trios. Illustrations drawn from the final boxed set are shown below in Figure 6.

Within the research and development of the Mind and Movement teaching resource, the many challenges included addressing how best to reformulate the CTT material developed with professional dancers into a simpler form appropriate for young students and their teachers. The recording procedures and notation tools we had developed for the professional dancers were too sophisticated and complex for younger students. Additionally, the teaching resource had to be used independently and in the absence of the tutorials and workshops given by researchers working on the earlier CTT project. In this simplification it was important to preserve, in a balanced way, essential aspects of the audit traces back into McGregor’s studio practice and into the field of cognitive science. The latter was a clear requirement to deliver the vision of seeking to augment studio practice with insights from science.

In response to these challenges, material in the lesson plans was organized by type of imagery, and example tasks were designed to work implicitly rather than explicitly with the more abstract intellectual properties derived from science. Rather than referring directly to making translations within the ‘loops’ or mental dialogues shown in Figure 6, strategies for varying and changing patterns of image were offered as a way to exercise and develop skills of the imagination. Similar concerns guided a selection of new principles derived from McGregor’s studio practice, and the vocabulary used on the individual principle cards was carefully selected to enable students at differing levels of sophistication to grasp the essence of what was required of them. In addition, aspects of the deeper ICS theory were described for teachers in accessible language that preserved key aspects of the theory alongside cues for guiding students’ attention. Examples of how lessons expressed imagery and creation tasks for a one kind of stimulus are illustrated in Figure 6.^15^ The CTT project demonstrates clear but separable intellectual audit traces back into the theoretical world of cognitive science, into arts research and into the creative skills of a renowned choreographer. In the next section we will consider how the concept of an audit trace can serve as a means to open up questions about what is learned in interdisciplinary collaboration and how it is learned.

## Discussion

It makes little sense to make unfounded inferences about how artists analyse information, assimilate it to their particular knowledge base, contextualize meanings in a production representation, resolve multiple constraints in the final performance synthesis or even how they evaluate and modify material as their process progresses. The PACT procedure has helped us to start populating some of the boxes in the Bridging Model in Figure 2 that make up this bigger picture in a grounded manner. This is important since we can only really use this filter when relevant substance is tied down in boxes at either end of each arrow. The labelling of the arrows (analyse, assimilate, contextualize, synthesize) linking the four boxes of the Bridging Model can be used to trigger questions and discussion about output produced by the PACT process. PACT provides many clear instances of audit traces for the decisions McGregor, or another choreographer, makes when planning a production, developing movement material for it, structuring it and integrating it with other dimensions of its ultimate performance. Two concrete examples of such reasoning coming from the PACT process for ATOMOS were outlined earlier, showing how the traces of the concepts of indivisibility and atoms described in Table 1 were informing the design phase involving creative iterations between the discovery and production representations in the Bridging Model.

Importantly, PACT captures audit traces of choreographic thinking occurring prior to entering the studio. In his preparatory or incubation phases for a new work, McGregor is ‘foraging’ across the right side of the Bridging Model in Figure 2. Ingredients of his discovery representation will eventually include, as possible translations into concrete tasks, a core set of attributes of movement he is researching and thinking about. In this phase McGregor may be reading material, visiting locations of relevance, watching films, conversing and noting ideas that arise out of serendipitous encounters. His own evolving practice also populates this ‘foraging’ landscape, as representations in the ‘landscape of production’ (left-hand box) are analyzed*,* filtered and then generically assimilated to these wider knowledge resources (right-hand box). In these pre-studio phrases he is also ‘designing’ key aspects of the production representation he is going to use – selecting and negotiating with collaborators for music, scenography, costume design and so on. Once in the studio his attention switches to other aspects. PACT provides a rich set of examples in the form of an audit trace showing when and why specific attributes appear or disappear from his production representation over the course of making (e.g. see deLahunta and Barnard forthcoming).

In the CTT project above, we revealed a different form of audit trace, showing a clear connection to the scientific theory as a basis for augmenting choreographic decision-making in the studio. From the CTT project we can illustrate how both science and arts resources can be collaboratively contextualized and how new discovery and production representations can come into existence through iterative design. These audit traces explicitly support McGregor’s interest ‘to feed into or build on aspects of choreographic thinking’ on the basis of deeper understanding, while their arrangement within the bridging model constitutes a road map for questioning and thinking about interdisciplinary collaboration. A number of observations are pertinent about the use of knowledge and the creation of new bridging representations that have interdisciplinary elements in their audit traces.

The CTT itself draws on two separate knowledge resources, one corresponding to a background commensurate with the scientific theory (Figure 5, ICS) and the other to the background of McGregor’s creative work. From there, the Bridging Models for science and art start to merge. As both artists and scientists collaborated in the design enterprise for CTT, the exchanges served to iteratively contextualize the relevant knowledge sources in a manner appropriate for professionals in both areas. While the experience sampling techniques used and written up for journal publication conformed to the requirements of scientific rigour (May et al. 2011), the particular form for systematic note taking we developed for the dancers was, as noted earlier, itself an element of a new discovery representation that emerged out of an iterative design process. Likewise the Mind and Movement resource for schools illustrated earlier is a production representation designed specifically in this case for pedagogy in a performance domain.

**Figure 7.**
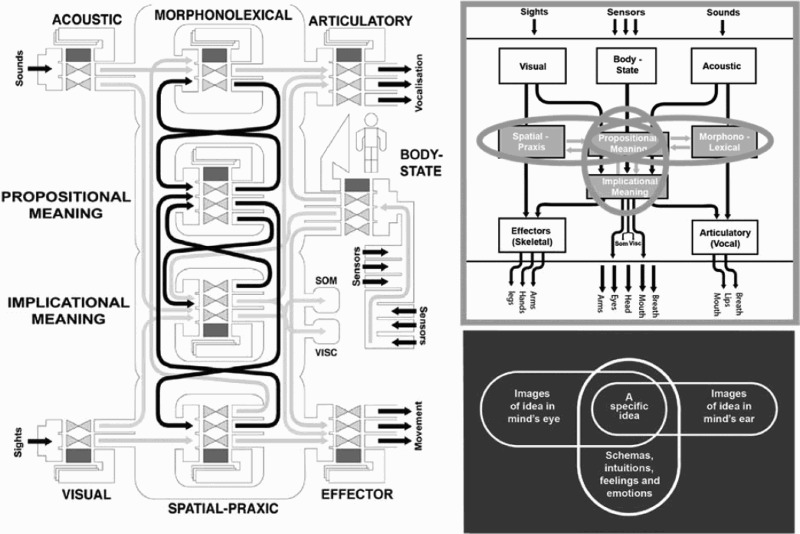
Three graphical representations of the ICS theory contextualized at alternate levels of specificity for work in basic science and its use with professional and student dancers. Each representation preserves the same fundamental relationship between spatial-praxic, auditory verbal and semantic imagery.

The audit trace for one of the bridging processes, the contextualization of the ICS scientific theory, is captured in Figure 7 with three alternative depictions of the same theory. The left-hand panel of this figure shows a very detailed representation of the theory for use by for professional scientists, the content of which need not be elaborated here except to note that there are three ‘loops’ realized as figure of eight pathways between the four central subsystems. The upper panel on the right-hand side is an annotated version of Figure 5. This is the way in which the same theory was contextualized as an application representation for use by professional dancers. The depiction in the lower right of this figure is the one we used one for dance teachers and their students. For science presentations the detailed components of the model and their interconnections are fully specified, many of which are omitted in the simpler characterizations. Note, for example, that in the final synthesis for the Mind and Movement resource, the representation of the theory shown in the lower right-hand panel was not only simplified but rendered in such a way as to make its appearance fully compatible and synthesized with the artistic design of the whole educational package of resources, rather than using the science convention of using arrows to notate information flows. Note that the annotation in the upper right panel of this figure highlights what is extracted into the panel in the lower right. The essential isomorphism or relationship among imagery systems is preserved across all three depictions, and with that, a systematic audit trace from science to artistic application is preserved. This is a clear example where the audit trace back into knowledge derived from psychology about imagery directly complements and blends with an equivalent audit trace for the incorporation of principles for transforming image content derived from McGregor’s practice.

## Conclusion

Following the description of the earlier Choreography and Cognition project it was noted that there was a clear trace of the involvement of the choreography domain in the reports of the science endeavours, but rather more of an ineffable ambiguity about the trace of the science in the choreography of AtaXia. In his discussion of the same project, the social anthropologist Leach (2006) also raised the question of what added value had or had not emerged from these projects. The subsequent work in developing PACT, the work with CTT and their characterization within Bridging Model framework enabled some more substantive claims to be proposed about audit traces and their links into what is learned or produced and how it is learned or produced.

The PACT process supports the specification of aspects of an artistic practice that distinguishes representations and processes that can be framed within the Bridging Model. The explicit representations, of course, are a way of thinking about what is learned from the projects, while analysis of the implicit processes that map between the boxes of the model tell us something about how it is learned. By executing this project and the CTT project we can gather together, in this case, elements of audit traces about global and local aspects of choreographic decision-making. We can also gather together audit traces of the contribution of science. Added value can then be seen in where we can justify evidence of complementarity or blending of these audit traces. The material products of the workshops underpinning the development of CTT for professional and student dancers all contain examples of added value where design decisions involved either complementarity or a blending of elements of the science with elements of the art. The added value of the work was not just confined to planned outputs. As noted earlier PACT was designed as a means to charting the detail of choreographic thinking. The idea that the procedure could be both a means of data capture and a ‘tool for thought’ for McGregor and for students was unforeseen at the outset of the work and was therefore an added bonus. More widely, the Bridging Model, and the PACT process in particular, offer routes to improving our understanding of what and how something is learned in the context of interdisciplinary work. They could be applied just as well to the interdisciplinary projects of other artists, scientists and scholars.

At the outset we used a quote of McGregor from 2008 that expressed an interest in knowing more about how choreographic decisions are made. McGregor’s own perspective has undoubtedly evolved, at least in part through this interdisciplinary research, to embrace more systematic processes:

This concept tracking has really allowed me to understand some of the mechanics of that. Actually, when is it important to understand when the design constraints affect perhaps some of the ways you’re going to work with structural form in the rehearsal process? When is it that all of a sudden two ideas which were seemingly opposed or very different parts of understanding coalesce to be able to grow into something that then becomes the centre of the thing that you’re working on? And I think the only way you can do that is to look at what some of those decisions are over time with some kind of methodology, with some kind of filter. (in McGregor and Barnard 2013)

## Footnotes

1 Random Dance was renamed Wayne McGregor | Random Dance in 2008. The organization is currently known as Studio Wayne McGregor and works are now performed by Company Wayne McGregor.

2 A handful of co-authored publications by artists working with scientists emerged from the project (e.g. delahunta, McGregor, and Blackwell 2004; deLahunta, Barnard, and McGregor 2009), but generally these could be seen as extensions of the shared space created by the interdisciplinary conversations stimulated by the project.

3 ‘the number and quality of outputs speak for themselves’ (Leach 2006, 448).

4 Notice that the graphic in the right hand panel is a reduced image of the theory described later in Figure 5.

5 and we have fleshed out elsewhere the bridging diagram for some aspects of the work of the Wayne McGregor | Random Dance and the research work carried out within R-Research (deLahunta and Barnard forthcoming).

6 The PACT process was used for two prior productions – FAR (Sadler's Wells premiere 2010) and UNDANCE (Sadler's Wells premiere 2011).

7 At least for ATOMOS, PACT material was shown in the Wellcome Collection exhibition just prior to the performance of ATOMOS where, once again a video can be viewed summarizing this particular project https://wellcomecollection.org/exhibitions/thinking-body-mind-and-movement-work-wayne-mcgregor-random-dance/?video=2.

8 Qualia is a concept in philosophy referring to a quality or property as perceived or experienced by a person.

9 A piece that premiered in 2008 celebrating Diaghilev and the hundredth anniversary of the Ballet Russe.

10 Characterized as a form of “embodied thinking” (Kirsh 2010).

11 Praxis in Latin means action.

12 This was divided between the Experimental Media and Performing Arts Centre (EMPAC) at the Rensselaar Polytechnic Institute in upstate New York and a residency at the Dance Centre at Columbia College in Chicago.

13 The three domains of cognitive skill involved (a) how to identify abstract properties driving their use of imagery, (b) sophisticated strategies for moving attention not only to material in their bodies and surrounding environment but also to alternative forms of mental imagery, and (c) strategies for translating imagery into movement.

14 While no formal ‘data’ was collected to assess the impact this resource had on outcomes with the professional dancers from the company, the reactions of both the dancers and McGregor were sufficient to consider its continued use of practical value.

15 A follow own project funded by the Leverhulme Trust involving a collaboration Plymouth University, Trinity Laban and Coventry University is now exploring extensions of the core resource to tertiary education: http://www.dancersmind.org.uk
